# Cadaveric Study for Intraoral Needle Access to the Infratemporal Fossa: Application to Posterior Superior Alveolar Nerve Block Technique

**DOI:** 10.7759/cureus.1761

**Published:** 2017-10-09

**Authors:** Joe Iwanaga, Emily Simonds, Rod J Oskouian, R. Shane Tubbs

**Affiliations:** 1 Seattle Science Foundation; 2 Swedish Neuroscience Institute; 3 Neurosurgery, Seattle Science Foundation

**Keywords:** maxilla, anatomy, nerve block, superior alveolar nerve, wisdom tooth, mandible, endodontics

## Abstract

The posterior superior alveolar (PSA) nerve block is, from an anatomical perspective, challenging because the mandibular ramus and the zygomatic process of the maxilla can interfere with the course of the needle. Dentists empirically know that shifting the patient’s mandible to the ipsilateral side can improve visibility and accessibility for such blocks. This cadaveric study aimed to establish anatomical evidence for the ipsilateral mandible shifting used in the PSA. The distance between the lateral most bulging point of the alveolar part of the maxilla and ipsilateral anterior border of the ramus of the mandible ranged from 1.4 to 6.9 mm with a mean of 4.3 ± 1.6 mm. This result supports the empirical technique of shifting the mandible ipsilaterally for PSA nerve block.

## Introduction

The posterior superior alveolar (PSA) nerve block is commonly used to anesthetize the upper molar teeth except for the mesiobuccal root of the upper first molar [1]. During endodontic treatment and oral surgery, the PSA nerve block is a common procedure. From an anatomical perspective, the PSA is challenging because the anterior border of the ramus of the mandible and the zygomatic process of the maxilla might interfere with the course of the needle. Based on anecdotal experiences, many dentists have learned to shift the patient’s mandible ipsilaterally during the PSA nerve blocks in order to improve visibility and easy advancement of the needle [2]. However, we could not find any anatomical studies indicating how much space is gained by shifting the mandible ipsilaterally. Therefore, this study aimed to establish such data via a cadaveric investigation.

## Materials and methods

Thirty sides from 15 fresh-frozen cadaveric Caucasian heads were used in this study. The specimens were derived from two males and thirteen females and the age at death ranged from 58 to 90 years old (mean age; 80.0 ± 9.3 years old). The maxillary molar status on each side was recorded as “molar group” (with one or more molars) or “non-molar group” (without molars). Prior to the measurements, palpation of the temporomandibular joint (TMJ) was performed to confirm that it was not dislocated.

**Figure 1:**
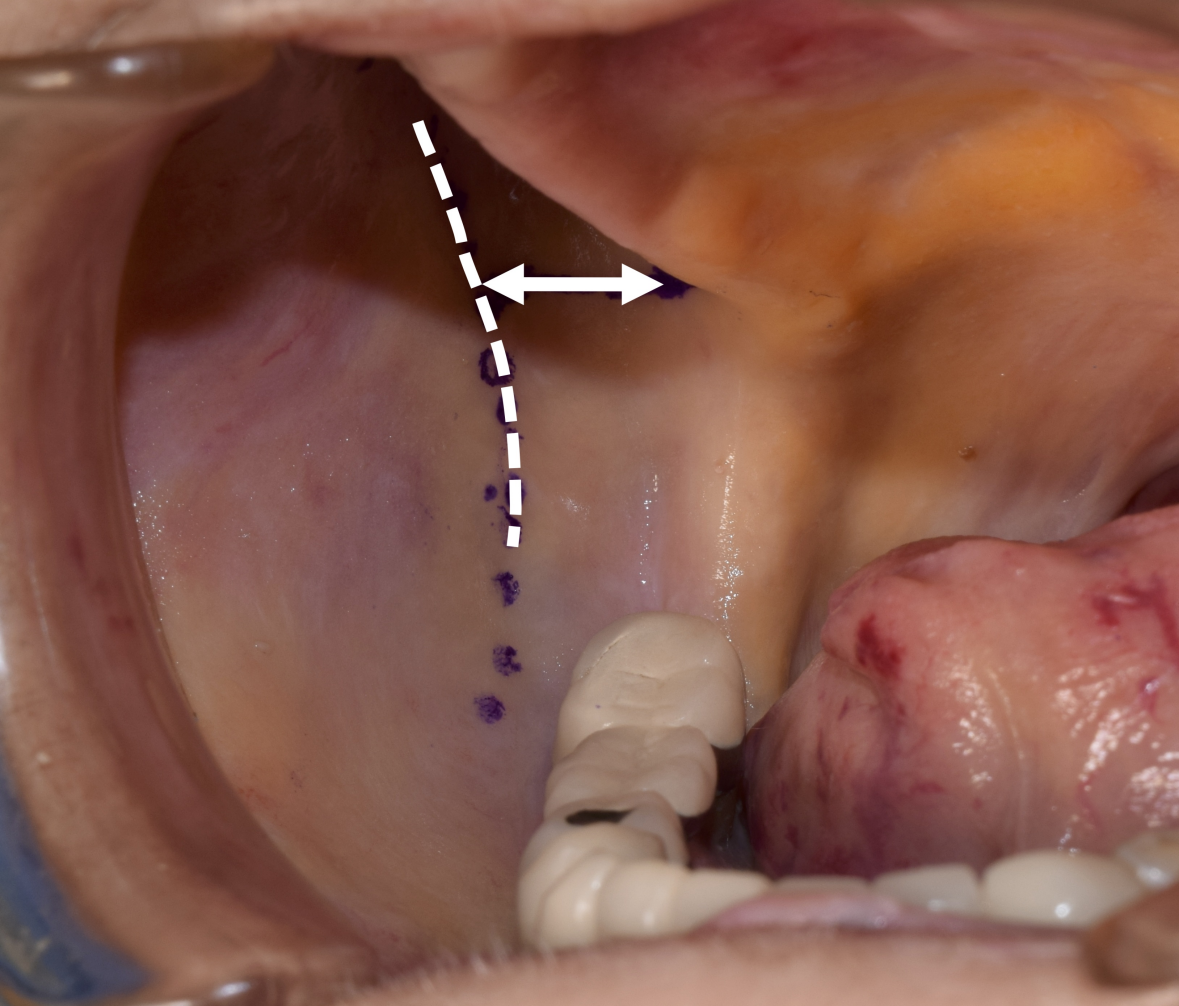
The figure demonstrates the narrow space (arrow) between the lateral most bulging point of the alveolar part of the maxilla and ipsilateral anterior border of the ramus of the mandible (dotted line) with the mouth opened maximally.

**Figure 2:**
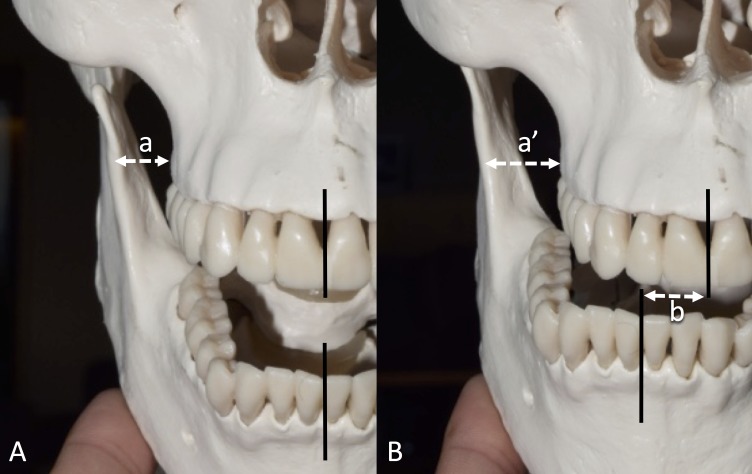
Skull model demonstrates the horizontal distance between the lateral most bulging point of the alveolar part of the maxilla to the anterior border of the ramus of the mandible ramus. The mandible is opened maximally (a) and is deviated ipsilaterally (a’). Note the horizontal change of the position of the central incisors along with the mandibular deviation (b). A: Mandible opened maximally, B: Mandible position deviated ipsilaterally.

First, the horizontal distance between the lateral most bulging point of the alveolar part of the maxilla to the anterior border of the ramus of the mandible was measured with the mandible opened maximally (a) (Figure 1) and deviated ipsilaterally (a') (as shown in Figure 2). The differences between these two numerical values (a'-a) were calculated. Secondly, the horizontal change of the position of the central incisors along with the mandible deviation (b) was measured (Figure 2). Next, the mean (a’-a)/ (b) was calculated. A horizontal reference line was used parallel to the line between the right and left pupils and a vertical line was made to the horizontal reference line. When the specimen had no central incisors, the anterior nasal spine and a vertical line began from the anterior nasal spine and were used as the midline. Lastly, the TMJ was incised to confirm that it was not dislocated.

Two clinical anatomists (J. I. and S. T.) performed all the measurements. The measurements were made with a microcaliper (Mitutoyo, Kanagawa, Japan) with a resolution of 0.01 mm and an accuracy value of ± 0.025 mm. The measurement was performed three times by each observer (for a total of six times for each measurement) and then averaged. The differences between the groups were evaluated using a t-test with a p-value < 0.05 which is considered significant. The present study protocol did not require approval by the ethics committees of our institutions and the work was performed in accordance with the requirements of the Declaration of Helsinki (64th World Medical Association (WMA) General Assembly, Fortaleza, Brazil, October 2013).

## Results

On all 30 sides, the distance (a’) was longer than the distance (a). As for the maxillary molar status, nine sides (30%) were categorized as the molar group and twenty-one sides (70%) were in the non-molar group. The distances (a) ranged from 8.9 to 15.9 mm with a mean of 12.2 ± 2.1 mm (11.9 ± 2.3 mm on the right side and 12.5 ± 2.0 mm on the left side). The distance (a’) ranged from 11.7 to 21.6 mm with a mean of 16.5 ± 2.6 mm (16.2 ± 2.8 mm on the right side and 16.8 ± 2.4 mm on the left side). The distance (a’-a) ranged from 1.4 to 6.9 mm with a mean of 4.3 ± 1.6 mm (4.3 ± 1.6 mm on the right side and 4.3 ± 1.7 mm on the left side). The distance (b) ranged from 4.3 to 13.6 mm with a mean of 8.1 ± 2.0 mm (7.7 ± 1.0 mm on the right side and 8.5 ± 2.5 mm on the left side) (Table 1). The measurements (a’-a)/ (b) ranged from 0.17 to 0.82 with a mean of 0.54 ± 0.20. The distance (a) in molar group ranged from 9.3 to 15.2 mm with a mean of 12.4 ± 2.3 mm, and in the non-molar group, ranged from 8.9 to 15.9 mm with a mean of 12.1± 2.1 (Table 2). There was no significant difference between molar group and non-molar group and the right and the left sides (p >0.05).

No injury to the nearby anterior border of the ramus of the mandible was observed on any of the cadaveric sides and the TMJ was not dislocated.

**Table 1:** The horizontal distance between the lateral most bulging point of the alveolar part of the maxilla to the anterior border of the ramus of the mandible were measured with the mandible opened maximally (a) and deviated ipsilaterally (a’). The differences between these two numerical values (a’-a) was calculated.

	Total		Right		Left	
	Range in mm	Mean in mm	Range in mm	Mean in mm	Range in mm	Mean in mm
(a)	8.9 - 15.9	12.2 ± 2.1	8.9 - 15.9	11.9 ± 2.3	9.5 - 15.3	12.5 ± 2.0
(a’)	11.7 - 21.6	16.5 ± 2.6	11.7 - 21.6	16.2 ± 2.8	12.6 - 20.6	16.8 ± 2.4
(a’-a)	1.4 - 6.9	4.3 ± 1.6	1.4 - 6.3	4.3 ± 1.6	1.4 - 6.9	4.3 ± 1.7
(b)	4.3 - 13.6	8.1 ± 2.0	5.9 - 10.1	7.7 ± 1.0	4.3 - 13.6	8.5 ± 2.5

**Table 2:** The maxillary molar status on each side was recorded as “molar group” (with one or more molars) or “non-molar group” (without molars). The difference of (a) between molar and non-molar groups was calculated.

	Molar (n=9)		Non-molar (n=21)	
	Range in mm	Mean in mm	Range in mm	Mean in mm
(a)	9.3 - 15.2	12.4 ± 2.3	8.9 - 15.9	12.1 ± 2.1

## Discussion

The PSA nerve block with some supplemental anesthesia is the first anesthetic choice for endodontic treatment in the patients with irreversible pulpitis in the upper molar teeth [3]. Since the PSA nerve block was described in detail in the year 1968 by Adatia [4], injection technique [5], anesthetics [6], and related anatomy [2] have been discussed. As this is a blind procedure, some complications have been reported such as ocular dysfunction and temporary blindness [7-10]. These complications might result from low visibility and the difficulty of access to the infiltration site caused by the coronoid process of the mandible and the zygomatic process of the maxilla. Empirically, the dentists know that shifting the mandible ipsilaterally improves visibility and accessibility. However, it is not known how much shifting is needed to make visibility and accessibility better. In the present study, the distance (a’-a) ranged from 1.4 to 6.9 mm with a mean of 4.3 ± 1.6 mm. To access the correct infiltration site, 4.3 mm could mean a large difference for the dentists. Also, (a’-a)/ (b) ranged from 0.17 to 0.82 with a mean of 0.54 ± 0.20, which might help to predict how much more visibility and accessibility is gained by shifting the mandible ipsilaterally.

The difficulty of the visibility and accessibility differ depending on various factors, such as the lateral angle of the coronoid process, thickness and angle of the ramus of the mandible, width of the maxillary arch, the position of the zygomatic process of the maxilla, and mobility of the TMJ. In this study, there was no significant difference in (a’-a) between the molar and non-molar groups. From a clinical perspective, however, the patients receive the PSA nerve block mostly for endodontic or other restorative treatments [2].

As for upper wisdom tooth extraction, the dentists have limited space to insert instruments such as dental elevators and dental extracting forceps correctly. Empirically, they know that shifting the mandible to the ipsilateral side makes extraction easier. The results of this study support the method of shifting the mandible and provides quantitative data for the upper wisdom tooth extraction using the PSA nerve block.

## Conclusions

The distance between the lateral most bulging point of the alveolar part of the maxilla and ipsilateral anterior border of the ramus of the mandible for the PSA nerve block could change with a range from 1.4 to 6.9 mm and a mean of 4.3 ± 1.6 mm. These results support the empirical technique of shifting the mandible ipsilaterally for PSA nerve block.
